# Reference intervals of mesenteric lymph node size according to lymphocyte counts in asymptomatic children

**DOI:** 10.1371/journal.pone.0228734

**Published:** 2020-02-10

**Authors:** Baohuan Cai, Huiming Yi, Wei Zhang

**Affiliations:** 1 Department of pediatrics, Tongji Hospital, Tongji Medical College, Huazhong University of Science and Technology, Wuhan City, Hubei Province, China; 2 Department of Medical Ultrasound, Tongji Hospital, Tongji Medical College, Huazhong University of Science and Technology, Wuhan City, Hubei Province, China; Chinese Academy of Medical Sciences and Peking Union Medical College, CHINA

## Abstract

There is no acknowledged reference interval of mesenteric lymph node size in healthy children, and the size criterion for mesenteric lymph node enlargement (MLNE) has long been controversial. This study aimed to explore the reference intervals of mesenteric lymph node size according to lymphocyte counts in asymptomatic children and to develop a more appropriate definition of MLNE. The asymptomatic children included were divided into five age strata: 2 to 3 yr; 3 to 4 yr; 4 to 5 yr; 5 to 6 yr; and 6 to 7 yr. Correlation analyses between lymphocyte counts and the long-axis diameter, short-axis diameter, and average diameter of the largest mesenteric lymph node (LMLN) were performed. A reference interval of the short-axis diameter of LMLN was established according to this correlation analysis in each age group. We also report a reference interval of lymphocyte count in each age group. This study revealed significant correlations between the short-axis diameter of LMLN and lymphocyte count in all age groups, as well as in subdivided boy groups and girl groups. The overall reference interval of the short-axis diameter of LMLN in children was 0.54 cm—1.03 cm, with mean value of 0.75 cm. This study supports the use of the short-axis diameter greater than 8–10 mm as the diagnostic criterion for primary mesenteric lymphadenitis based on the presence of a cluster of three or more mesenteric lymph nodes and in the absence of other abnormalities.

## Introduction

Acute, chronic, or recurrent abdominal pain is a very common symptom in children, occasionally affecting the daily life of these patients [1–3]. Ultrasound examination of the abdomen is frequently performed in pediatric patients with abdominal pain [4], and a high prevalence of mesenteric lymph node (MLN) in the right lower abdominal quadrant has been previously reported [5, 6]. The diagnosis of lymph node enlargement by medical imaging is performed based on the size criterion. Unfortunately, the size criterion for mesenteric lymph node enlargement (MLNE) has long been controversial [2, 7], and there is no consensus regarding the definition of enlargement of mesenteric lymph node [8].

The current definition of MLNE is a cluster of three or more lymph nodes with short-axis diameter ≥ 5 mm [2, 9]. However, a MLN greater than 5 mm in the short-axis diameter is a common ultrasound finding in children during clinic examination and is frequently observed in asymptomatic children [7]. Moreover, a high percentage (54%) of false-positive was reported using a threshold of ≥ 5 mm in the short-axis diameter for MLNE [2]. Therefore, the criterion for MLNE as defined by the short-axis diameter ≥ 5 mm is considered inappropriate [2]. Some researchers even consider enlarged MLNs as a relatively common and non-specific finding [10], and argue about the importance of MLNE in children [7]. Other researchers have suggested that current size criterion for determining MLNE in children has an obvious overlap with the size interval of normal mesenteric lymph nodes, and using short-axis diameter greater than 8 mm might be a more appropriate clinical definition of pathologic mesenteric lymph node in children [2, 7, 11, 12]. In addition, there is no acknowledged reference interval of MLN size in healthy children. This study aimed to analyze the reference intervals of mesenteric lymph node size according to lymphocyte counts in asymptomatic children and to explore a more appropriate definition of MLNE.

## Materials and methods

### Patients

A total of 177 asymptomatic children, referred for abdominal ultrasound examinations for follow-up of mild hydronephrosis between October 2017 and September 2018, were included for MLN examinations. All of the children included were recruited from the outpatient clinic of the Department of Pediatrics at Tongji hospital, Tongji medical college, Huazhong University of Science and Technology.

Children with clusters of mesenteric lymph nodes, defined as the presence of 3 or more closely positioned MLNs, were included in this study. Follow-up of these children was done up to 2 months after the ultrasound examinations through contact with their parents or pediatricians. The exclusion criteria included congenital anomalies, intussusception, appendicitis, tuberculosis, lymphoma, gastrointestinal perforation, bloody purulent stool, as well as children with tumors or systemic diseases. Children with a medical history and other conditions that affected lymphocyte counts four weeks prior to ultrasound examinations were also excluded from this study. Of 177 children, 26 and 15 pediatric patients were excluded from this study because of urinary infection and respiratory infection four weeks prior to the ultrasound examinations, respectively. In addition, four children were also excluded due to failure of follow-up after the ultrasound examinations. Finally, a total of 132 children were enrolled in this study. The children enrolled in this study were divided into five age strata: 2 to 3 years old (n = 25, girl = 12 and boy = 13); 3 to 4 years old (n = 36, girl = 18 and boy = 18); 4 to 5 years old (n = 31, girl = 12 and boy = 19); 5 to 6 years old (n = 23, girl = 13 and boy = 10); and 6 to 7 years old (n = 17, girl = 9 and boy = 8).

The present study was approved by the Ethics Committee of Tongji Hospital and was performed in accordance with principles in the declaration of Helsinki (IRB ID: TJ-C 20170315). Written informed consent was obtained from a parent of the study participants prior to participation. All data were anonymized during analysis.

### Ultrasonography

All the ultrasound examinations were performed by the same experienced sonographer (WZ) with a GE LOGIQ E9 ultrasound machine (GE Healthcare, Wauwatosa, WI, USA) when each child was enrolled in this study. After scanning of the whole abdomen and pelvis with a curved 4 MHz transducer, mesenteric lymph node evaluations were performed with a linear 9 MHz transducer. The short-axis diameter and the long-axis diameter of the largest mesenteric lymph node (LMLN) were recorded for each child. Then the average diameter, defined as (the short-axis diameter + the long-axis diameter) / 2, as well as the ratio of the long-axis diameter to the short-axis diameter were calculated.

### Lymphocyte count

An EDTA-anticoagulated whole-blood specimen from each child enrolled in this study was collected on the same day of the ultrasound examination. All blood samples were collected and analyzed by the clinical laboratory of Tongji hospital on that same day. Lymphocyte count of each child was provided post blood sample analysis using a Sysmex KX-21 fully automated Hematology Analyzer (East Asia Co., Japan).

### Statistical analysis

SPSS-19 (SPSS Inc., Chicago, Illinois, USA) was used for all statistical analyses. Continuous variables were presented as the mean ± standard deviation (SD) and were analyzed using Student’s t-test. The correlation analyses between size of LMLN and lymphocyte count were performed using the Pearson rectilinear correlation analysis and linear regression analysis. The threshold level for statistical significance was p<0.05.

## Results

### Clinical features

**Fig 1:**
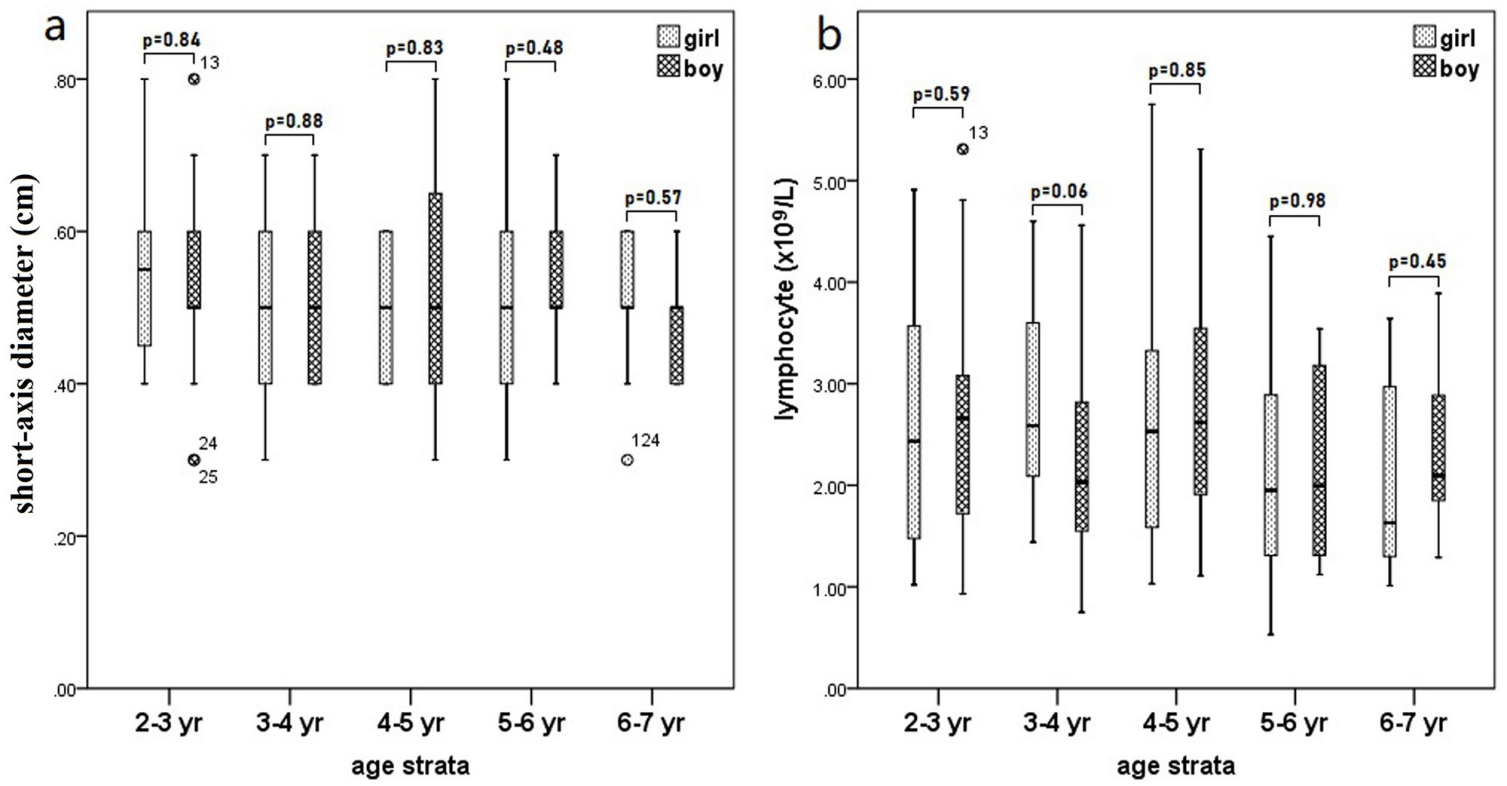
The short-axis diameters of the largest mesenteric lymph nodes (a) and lymphocyte counts (b) in each age group of children included in this study.

A total of 132 children were enrolled in this study. Of these children, 64 were girls and 68 were boys, with median age of 4.2 years old (2.0–7.0 years old). The characteristics of children included were presented in Table 1. No statistical significance existed between the girl and boy groups in the short-axis diameter of LMLN or in the lymphocyte count of each age group (Fig 1).

**Table 1:** Characteristics of children included.

	Gender	2–3 yr	3–4 yr	4–5 yr	5–6 yr	6–7 yr
Number of Girl/Boy		12/13	18/18	12/19	13/10	9/8
Height (cm)	Girl	92.42±5.24	101.0±4.14	106.08±6.26	115.23±5.06	119.67±4.74
	Boy	92.00±4.57	99.44±4.37	108.63±6.80	112.30±6.50	119.63±5.57
Weight (kg)	Girl	13.75±1.80	16.83±2.05	17.96±2.07	22.15±3.80	22.72±2.25
	Boy	13.12±1.32	15.86±1.85	18.05±2.94	20.20±2.89	21.63±3.00
Long-axis diameter^#^ (cm)	Girl	1.11±0.29	1.03±0.24	1.07±0.17	1.02±0.25	1.06±0.19
	Boy	1.06±0.20	1.05±0.13	1.08±0.25	1.09±0.15	1.05±0.17
Short-axis diameter^#^ (cm)	Girl	0.55±0.12	0.51±0.13	0.50±0.08	0.50±0.15	0.50±0.09
	Boy	0.54±0.14	0.51±0.08	0.51±0.14	0.54±0.08	0.48±0.07
Average diameter^#^ (cm)	Girl	0.83±0.21	0.77±0.18	0.78±0.12	0.76±0.19	0.78±0.14
	Boy	0.80±0.17	0.78±0.10	0.79±0.19	0.82±0.11	0.76±0.11
Ratio	Girl	1.98±0.15	2.05±0.26	2.14±0.19	2.09±0.33	2.13±0.22
	Boy	2.02±0.27	2.11±0.27	2.17±0.37	2.03±0.15	2.21±0.15
Lymphocyte count (x10^9^)	Girl	2.59±1.26	2.78±0.84	2.73±1.42	2.20±1.07	2.01±0.90
	Boy	2.88±1.29	2.17±0.98	2.82±1.21	2.21±0.89	2.36±0.83

Ratio, long-axis diameter/short-axis diameter; diameter#, diameter of the largest mesenteric lymph node

### The correlation analyses of lymphocyte count and size of LMLN

Results of the correlations between lymphocyte count and size of LMLN were shown in Table 2.

**Table 2:** Correlation analyses of lymphocyte count and size of the largest mesenteric lymph node.

Size	Gender	2–3 yr	3–4 yr	4–5 yr	5–6 yr	6–7 yr
Short-axis diameter	Girl	p = 0.013*	p = 0.020*	p = 0.033*	p = 0.035*	p = 0.048*
	Boy	p = 0.046*	p = 0.017*	p = 0.018*	p = 0.011*	p = 0.039*
Long-axis diameter	Girl	p = 0.044*	p = 0.062	p = 0.019*	p = 0.082	p = 0.086
	Boy	p = 0.218	p = 0.034*	p = 0.031*	p = 0.035*	p = 0.035*
Average diameter	Girl	p = 0.020*	p = 0.037*	p = 0.017*	p = 0.052	p = 0.060
	Boy	p = 0.117	p = 0.015*	p = 0.022*	p = 0.018*	p = 0.030*
Ratio	Girl	p = 0.723	p = 0.206	p = 0.943	p = 0.193	p = 0.633
	Boy	p = 0.059	p = 0.344	p = 0.306	p = 0.553	p = 0.716

Ratio, long-axis diameter / short-axis diameter,

* p<0.05

**Fig 2:**
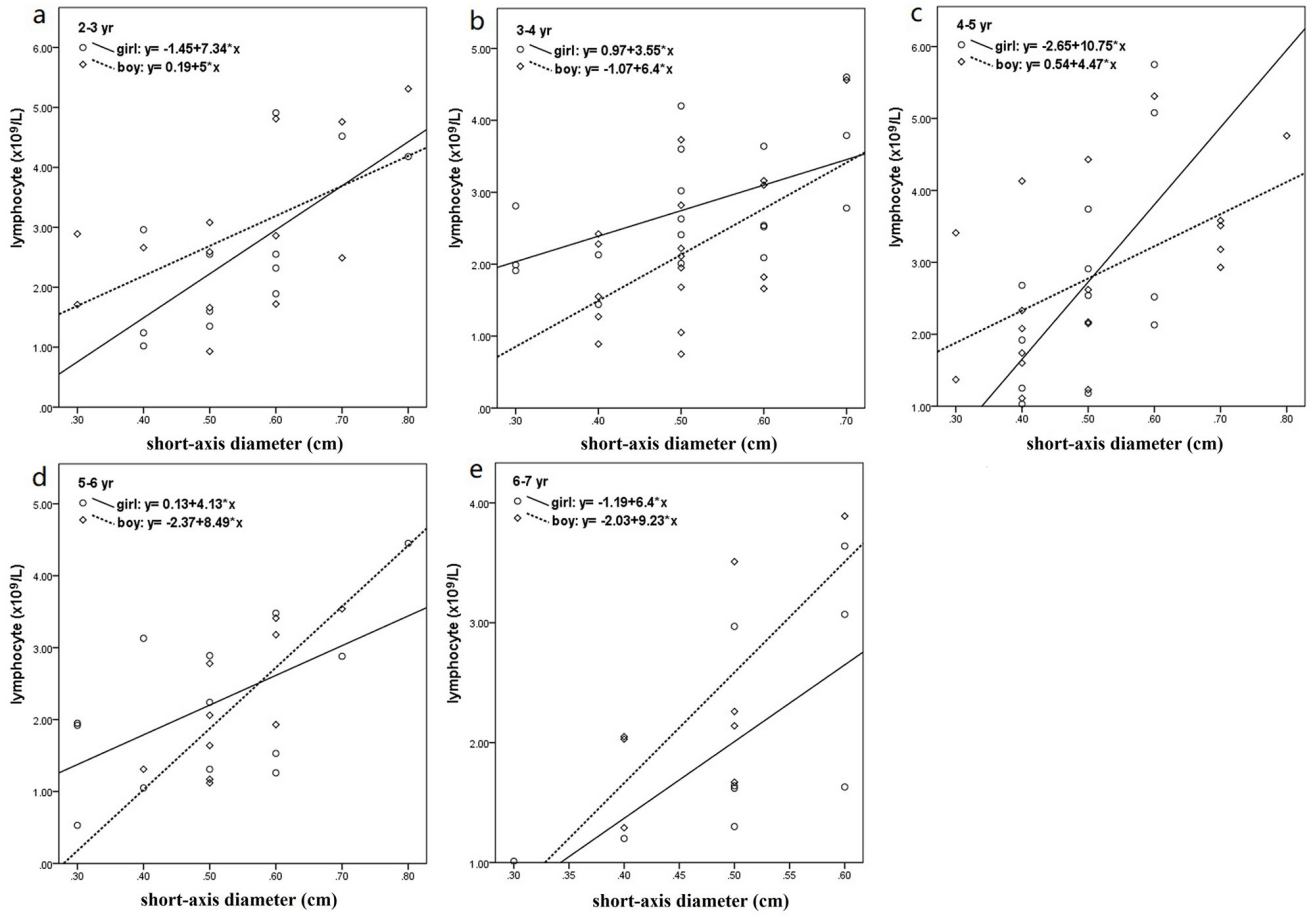
Results of fitting lines of lymphocyte counts with the short-axis diameters of the largest mesenteric lymph nodes for 2–3 yr group (a), 3–4 yr group (b), 4–5 yr group (c), 5–6 yr group (d), 6–7 yr group (e).

There were significant correlations between lymphocyte count and the short-axis diameter of LMLN for all of the five age groups, and the correlation coefficients were as follows: 0.589 (p = 0.013) for girl and 0.562 (p = 0.046) for boy in 2–3 yr group, 0.542 (p = 0.020) for girl and 0.554 (p = 0.017) for boy in 3–4 yr group, 0.617 (p = 0.033) for girl and 0.536 (p = 0.018) for boy in 4–5 yr group, 0.587 (p = 0.035) for girl and 0.760 (p = 0.011) for boy in 5–6 yr group, 0.670 (p = 0.048) for girl and 0.733 (p = 0.039) for boy in 6–7 yr group, respectively. The fitting lines are illustrated in Fig 2.

Significant correlations between lymphocyte count and the long-axis diameter and/or the average diameter of LMLN were only found in some of the subdivided groups (Table 2). No correlation between lymphocyte count and the ratio of the long-axis diameter to the short-axis diameter was found in this study (Table 2).

### Estimated values of the short-axis diameter intervals of LMLN

The reference intervals of lymphocyte counts in healthy Chinese children, shown in Table 3, had been previously reported by Jia LT [13]. All of the lymphocyte counts in the children enrolled in this study were lower than these reference intervals. Estimated values for the short-axis diameter intervals of LMLN in each subdivided group were calculated (Table 4) based on the correlations and fitting lines between lymphocyte count and the short-axis diameter of the mesenteric lymph node, along with the reference intervals of lymphocyte counts cited above. The overall estimated interval of the short-axis diameter of LMLN in all the children included in this study was 0.54 cm—1.03 cm, with mean value of 0.75 cm.

**Table 3:** Reference intervals of lymphocyte counts in healthy Chinese children reported by Jia LT [8].

Gender	Lymphocyte count, mean (minimum-maximum) (x109/L)				
	2–3 yr	3–4 yr	4–5 yr	5–6 yr	6–7 yr
Boy	4.34(3.08–6.52)	4.13(2.73–5.97)	3.59(2.75–5.19)	3.74(2.61–4.98)	3.24(2.19–4.54)
Girl	4.95(3.34–7.49)	3.98(2.63–5.50)	3.74(2.73–5.33)	3.72(2.56–4.99)	3.25(2.17–4.43)

**Table 4:** Estimated values of the short-axis diameter intervals of the largest mesenteric lymph node according to fitting lines and reference intervals of lymphocyte counts.

Age	Maximum (cm)		Mean (cm)		Minimum (cm)	
strata	Girl	Boy	Girl	Boy	Girl	Boy
2–3 yr	1.22	1.27	0.87	0.83	0.65	0.58
3–4 yr	1.28	1.10	0.85	0.81	0.47	0.59
4–5 yr	0.74	1.04	0.59	0.68	0.50	0.49
5–6 yr	1.18	0.87	0.87	0.72	0.59	0.59
6–7 yr	0.88	0.71	0.69	0.57	0.53	0.46
Average	1.06	1.00	0.77	0.72	0.55	0.54
Girl vs Boy	p = 0.675		p = 0.509		p = 0.909	
	Total average = 1.03		Total average = 0.75		Total average = 0.54	

## Discussion

Ultrasonography is considered the most appropriate imaging approach for the evaluation of mesenteric lymph nodes in children [7, 14–17]. However, there is no acknowledged reference interval of MLN size in healthy children, and the diagnostic criterion for MLNE has long been controversial [2, 7]. The current definition of MLNE as greater than 5 mm in the short-axis diameter was reported to result in a high false positive rate [2], which will lead to unnecessary treatment and follow-up. Therefore, the reference intervals of mesenteric lymph node size and a more appropriate definition of MLNE are urgently needed in clinical practice.

In the present study, correlation analyses of LMLN size with lymphocyte count were performed in order to establish the reference intervals of mesenteric lymph node size in asymptomatic children. The mesenteric lymph nodes increase in size during childhood until approximately 6 years old [2, 12]. Lymphocyte counts also vary during childhood, and age is a crucial and influential element in lymphocyte count variations [13, 18]. Therefore, the children included in this study were divided into five age strata: 2–3 yr group, 3–4 yr group, 4–5 yr group, 5–6 yr group and 6–7 yr group, and each age group was further subdivided into boy groups and girl groups.

This study revealed significant correlations between the short-axis diameter of LMLN and lymphocyte count in all age groups, as well as in subdivided boy groups and girl groups (Table 2). The long-axis diameter and average diameter of LMLN were only correlated with lymphocyte count in some of the subgroups. The ratio of the long-axis to short-axis diameter of LMLN was not associated with lymphocyte count in any age group. Therefore, the short-axis diameter of LMLN can be applied for the evaluation of each age group, rather than the long-axis diameter or average diameter. This result is consistent with the concept of using the short-axis diameter as the diagnostic parameter in existing criteria [2, 7, 9, 11, 12].

Except for mild hydronephrosis, the children enrolled in this study were healthy, although the values of lymphocyte count obtained in this study were lower than the established reference intervals [13], which may due to the small sample number. According to the correlation of the short-axis diameter of LMLN with lymphocyte count and fitting line in each age group, and by using the established reference intervals of lymphocyte counts for healthy Chinese children [13], the reference interval of the short-axis diameter of LMLN was calculated in each age group (Table 4). The overall reference interval of the short-axis diameter of LMLN in children was 0.54 cm—1.03 cm, with mean value of 0.75 cm. According to the estimated reference intervals of LMLN size, this study supports the use of the short-axis diameter greater than 8–10 mm as the diagnostic criterion for MLNE, which is consistent with the suggestion of using the short-axis diameter greater than 8 mm as the definition of MLNE [2, 7, 11, 12]. Compared with the definition of MLNE as short-axis diameter ≥ 5 mm, the criterion of MLNE defined as short-axis diameter greater than 8–10 mm should reduce the false positive rate and avoid unnecessary treatments.

Mesenteric lymph node enlargement can be associated with primary etiology, or can be secondary to various infectious, malignant, or inflammatory disorders [6, 8, 19]. In the absence of other abnormalities, enlarged mesenteric lymph nodes have been attributed to primary mesenteric lymphadenitis [2, 5]. With the estimated reference intervals of the size of LMLN established in this study, the short-axis diameter greater than 8–10 mm could be used as the diagnostic criterion for primary mesenteric lymphadenitis based on the presence of a cluster of three or more mesenteric lymph nodes and in the absence of other abnormalities [2, 5, 7, 19]. Furthermore, the diagnosis of mesenteric lymphadenitis relies on imaging, medical history, and clinical features [20–23]. It is particularly important that the identification of MLNE should not preclude the search for additional abdominal or pelvic abnormalities, as the erroneous attribution of enlarged mesenteric lymph nodes to mesenteric lymphadenitis has a potential risk of missing other acute diseases, such as acute appendicitis, intussusception, torsion of the ovary and so on, which require emergent surgical treatments [2, 8, 24]. In addition to primary mesenteric lymphadenitis, the reference intervals of MLN size and the definition of MLNE established in this study have the potential to be applied to various MLN disorders. Nevertheless, since 80.8% of positive mesorectal lymph nodes in rectal neuroendocrine tumors and 70.4% of positive mesorectal lymph nodes in rectal cancer were reportedly <5 mm in diameter [25, 26], implying small sizes of metastatic lymph nodes for rectal malignancy. Therefore, although rectal malignancy rarely occurs in children and mostly affects mesorectal lymph nodes, it’s logical that using size alone as a diagnostic criterion for mesenteric lymph node metastasis should be applied with caution.

This study has several limitations. Because of the limited number of children and single center design, these results should be interpreted with caution. The children included in this study were all asymptomatic. At present, there is no recognized normal value of mesenteric lymph node size for asymptomatic children [2], so the reference intervals of LMLN estimated in this study may only be suitable for asymptomatic children, and further study is needed to establish reference intervals of mesenteric lymph nodes in children with abdominal pain. In this study, the reference intervals of LMLN were established based on the reported reference values of lymphocyte counts in normal Chinese children, and the children enrolled in this study also came from China. In addition, since only reference values of lymphocyte counts in children younger than seven years old were reported [13], and only 2–7 years old children were enrolled during the study period, so the results of the present study may only apply to Chinese children of 2–7 years old. Therefore, studies involving different ethnic groups as well as a wider age distribution are needed to refine the results obtained in the present study. Although a positive correlation between the short-axis diameter of LMLN and lymphocyte count in children was found in this study, the biological mechanism was not revealed. Recently, in addition to the binding of Platelet Selectin Glycoprotein Ligand 1 (PSGL1) expressed on activated T cell to P-selectin expressed on activated endothelia and platelets, the binding of P-selectin-Ligand-2 (PSL2) to both L-selectin expressed on activated T cells and P-selectin was also found engaged in movement of leukocytes from blood into lymph nodes in mice [27]. However, further studies are still needed to confirm the fundamental process of T cell recruitment and lymph node enlargement *in vivo* in the future.

## Conclusion

In asymptomatic children, the short-axis diameter of LMLN was significantly correlated with lymphocyte count. The overall estimated interval of the short-axis diameter of LMLN in children was 0.54 cm—1.03 cm, with mean value of 0.75 cm. This study supports the use of the short-axis diameter greater than 8–10 mm as the diagnostic criterion for primary mesenteric lymphadenitis based on the presence of a cluster of three or more mesenteric lymph nodes and in the absence of other abnormalities.
